# Unveiling the Burden of Leukemia in Eritrea (2010–2020): Chronic Leukemia Stability, Rising ALL Incidence, and the Enigma of CML in a Low‐Resource Setting

**DOI:** 10.1002/cnr2.70203

**Published:** 2025-06-16

**Authors:** Daniel Mebrahtu Abraha, Samuel Tekle Mengistu, Efriem Ghirmay, Eden Gebresilassie, Ghirmay Embaye Zerat, Rahwa Kokob, Asmeret Tesfazghi, Solomon Negash, Tsega Daniel, Salih Mohammed, Oliver Okoth Achila

**Affiliations:** ^1^ Hematology National Health Laboratory Asmara Eritrea; ^2^ Nakfa Hospital Ministry of Health, Northern Red Sea Branch Nakfa Eritrea; ^3^ Quality Control National Food and Medicine Administration Asmara Eritrea; ^4^ Hematology Orotta National Referral Hospital Asmara Eritrea; ^5^ Unit of Clinical Laboratory Science Orotta College of Medicine and Health Sciences Asmara Eritrea

**Keywords:** ALL, AML, CLL, CML, Eritrea, hematological malignancy, incidence, leukemia, trend

## Abstract

**Background and Aims:**

Little research has been conducted on the epidemiology of leukemia in Eritrea. In this retrospective study, we evaluated the burden and trends of acute lymphoblastic leukemia (ALL), acute myelogenous leukemia (AML), chronic myelogenous leukemia (CML), chronic lymphocytic leukemia (CLL), and overall leukemia in Eritrea.

**Methods and Results:**

An audit of leukemia cases recorded in laboratory logbooks at the National Health Laboratory and Orotta Referral and Teaching Hospital between January 2010 and December 2020 was performed. In addition to leukemia subtypes, variables such as age, sex, and year of incidence were retrieved. Relevant estimates assessed included crude incidence rates (CIR), age‐standardized rates (ASIR), and estimated annual percentage change (EAPC). In total, 372 confirmed cases of leukemia were recorded between 2010 and 2020. The median (interquartile range [IQR]) age, maximum–minimum age, and male‐to‐female ratio were as follows: 48 years (24.5–60 years), 2–91 years, and 210:161 (1.3:1), respectively. The estimated all‐age CIR and ASIR over the study period were 9.22 per 100,000 and 30.1 per 100,000, respectively. Analysis of cumulative (2010–2020) CIR per 100,000 (ASIR per 100 000) for ALL, AML, CLL, and CML were as follows: 2.01 (3.87); 0.94 (2.38); 2.94 (15.37); and 3.61 (24.03). Additionally, the median (IQR) age differed significantly across leukemia subtypes: ALL (23.0 years, IQR: 10.0–39.0); AML (30 years, IQR: 20–56 years); CLL (59.0 years, IQR: 40.75–66.75 years); and CML (49 years, IQR: 39.25–60 years), *p* value (Kruskal–Wallis) < 0.05. The proportion of leukemia types did not differ significantly between males and females. Unlike other leukemia subtypes, the evaluation of ALL's EAPC demonstrated that the incidence of leukemia increased over time, 21.9 (95% CI: 3.1–44.1), *p* value = 0.025.

**Conclusions:**

The burden of leukemia in Eritrea remained relatively stable. However, due to underreporting and underdiagnosis, we believe the true burden of leukemia is likely higher. Furthermore, an upward trend in the burden of ALL was observed. Lastly, expanding diagnostic services to other sub‐zones, establishing a national cancer registry, and prioritizing research remain critical in Eritrea.

## 1 Introduction

Leukemia encompasses a heterogeneous group of hematologic malignancies originating in the bone marrow and blood‐forming tissues [1, 2]. These cancers are characterized by the uncontrolled proliferation of immature blood cells, which disrupt normal hematopoiesis and can infiltrate other organs [3, 4]. The classification of leukemia is based on cell lineage and disease progression. Cell lineage distinguishes between lymphoid leukemias and myeloid leukemias [4, 5], while disease progression categorizes leukemia as either acute or chronic [5]. This fundamental classification framework guides diagnostic approaches, treatment strategies, and prognostic assessments.

The understanding and classification of leukemia have undergone a remarkable evolution [5, 6, 7, 8, 9, 10, 11], as have therapy and prognosis [12]. Early classifications relied heavily on morphological features observed under a microscope, combined with clinical presentations. The French‐American‐British (FAB) classification system, for instance, utilized morphologic and cytochemical criteria to categorize acute leukemias [5, 6]. The identification of specific genetic mutations, chromosomal translocations, and gene expression profiles has led to the development of more refined classification systems, such as those published by the World Health Organization (WHO) [7, 8, 9, 11].

Leukemia constitutes a substantial global health burden, impacting millions worldwide. The incidence, prevalence, and mortality rates of leukemia vary significantly across regions and populations [13]. Several studies use metrics like the crude incidence rate (CIR; an unadjusted snapshot of disease occurrence), age‐standardized rates (ASR; accounting for differences in population age structures to enable meaningful comparisons across regions and time periods), and estimated annual percentage changes (EAPC; a statistical measure of annual trends that highlights whether leukemia incidence is increasing or decreasing and provides valuable insights for guiding healthcare planning, resource allocation, and policy formulation) to accurately characterize these trends [14]. Globally, new cases of leukemia increased from 311 648 in 1990 to 461 423 in 2021, and are expected to rise to more than half a million in 2050 [13]. Of the new cases in 2021, 57.2% were male, 70.8% were middle‐aged or older (> 45 years), and acute myeloid leukemia (31.4%) remained the most common subtype [13, 15, 16, 17]. Leukemia incidence declined significantly from 6.9 to 5.6 per 100 000, mortality from 5.6 to 3.9, and disability‐adjusted life years (DALYs) from 266.3 to 136.9 between 1990 and 2021 [13].

The burden of leukemia in Africa, particularly Sub‐Saharan Africa, presents unique challenges. While precise figures remain elusive due to limited cancer registration and data collection infrastructure [15, 18, 19, 20, 21, 22], available evidence suggests a significant disparity in leukemia incidence, prevalence, and mortality compared to high‐income countries [22]. In 2021, the burden of leukemia was highest in East Africa, with 8286 adult cases (ASIR 4.5) and 2954 pediatric cases (ASIR 1.6), compared to the lowest in West Africa, with 3612 adult cases (ASIR 2.3) and 790 pediatric cases (ASIR 0.47). Middle Africa and Southern Africa showed moderate burdens, with adults reporting 2196 cases (ASIR 3.1) and 1981 cases (ASIR 5.0), respectively [23].

Currently, there is a paucity of published research on the epidemiology of leukemia specifically in Eritrea. A thorough search of available literature databases reveals a significant gap in the comprehensive epidemiological characterization of leukemia in this nation. The existing data fail to provide reliable estimates of CIR, ASR, and EAPC, which are critical for accurate assessment of leukemia trends and for effective public health planning [24, 25, 26].

This study will shed light on the unique epidemiological characteristics of leukemia in Eritrea and inform the development of context‐specific strategies for prevention and control.

## 2 Methods

### 2.1 Study Design and Settings

An audit of leukemia cases presenting to the National Health Laboratory (NHL) and Orotta Referral and Teaching Hospital (ORTH) between January 2010 and December 2020 was performed. Currently, these two facilities are the only centers in Eritrea capable of diagnosing leukemia. NHL serves as a referral laboratory for all hospitals in the country, while Orotta Hospital functions as the national referral hospital. Therefore, all suspected leukemia specimens in Eritrea are processed at these two facilities for confirmation and further decision on managing the cases.

### 2.2 Study Population

**FIGURE 1:**
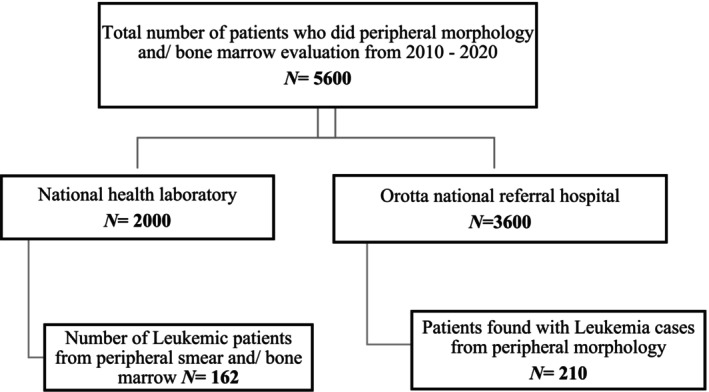
Flow diagram of study participants from National Health Laboratory, Hematology department: 2010–2020.

The study population included patients suspected of having leukemia in facilities across Eritrea and referred to NHL or ORTH for diagnosis confirmation. All Eritrean patients recorded in the registry with a diagnosis of leukemia, regardless of gender or age, between January 1, 2010 and December 31, 2020 were included in this study. Non‐Eritrean patients with leukemia and registered cases missing principal data (e.g., leukemia status or subtypes) were excluded. Overall, as shown in Figure 1, a total of 5600 samples were processed at the two facilities from 2010 to 2020 (2000 at NHL and 3600 at ORTH).

### 2.3 Data Collection Procedure

Data for the study were collected from laboratory records at the two institutions. Key variables included age at diagnosis, sex, and year of diagnosis. While the type of leukemia diagnosed was recorded, information on cancer stage and eventual outcomes was not recorded. To maintain confidentiality and minimize duplication, codes were used. The inclusion criteria encompassed all male and female patients of all ages diagnosed with leukemia in Eritrea. To ensure comprehensiveness and minimize missing information, the collected data were crosschecked by three different enumerators.

### 2.4 Operational Definitions

#### 2.4.1 Diagnosis of Leukemia

The International Classification of Diseases (ICD) definitions of disparate leukemia subtypes developed by the World Health Organization (WHO) were adopted in this study. Relevant codes include AML (C92.0–C92.02, C92.3–C92.62, C93.0–C93.02, C94.0–C94.02, C94.2–C94.22), ALL (C91.0–C91.02), CML (C92.1–C92.12), and CLL (C91.1–C91.12) [27]. Diagnosis followed established cytological protocols. In our center, leukemia classification relies solely on microscopic examination due to the absence of advanced diagnostic tools such as flow cytometry, immunohistochemistry, and molecular testing. As such, we adhere to the traditional morphological classification system, which is based on cellular appearance, cell size, nuclear morphology, and the percentage of blasts in peripheral blood and/or bone marrow smears. This approach allows us to classify leukemia into the four major types: acute lymphoblastic leukemia (ALL), acute myeloid leukemia (AML), chronic lymphocytic leukemia (CLL), and chronic myeloid leukemia (CML). This system is consistent with historical classification methods (early 1900s) that laid the foundation for modern leukemia classification.

#### 2.4.2 Parameters Used in Our Center

1. *Blast percentage for acute leukemia* (*ALL* & *AML*)
  - Following WHO criteria, leukemia is classified as “acute” if the percentage of blasts in the peripheral blood or bone marrow is ≥ 20%.
  - Blasts are identified and counted manually using Wright‐Giemsa‐stained smears.
2. *Smudge cells for chronic lymphocytic leukemia* (*CLL*)
  - CLL diagnosis is supported by the presence of smudge cells, which are fragile lymphocytes that rupture during smear preparation.
  - Smudge cells are a common morphological feature of CLL, though not pathognomonic. Their presence, along with an increase in mature lymphocytes, supports the diagnosis of CLL.
3. *Age lineage for leukemia types*
  - ALL: Predominantly affects children and young adults, though it can also occur in older adults.
  - AML: Primarily seen in adults and older populations, with incidence increasing with age.
  - CLL: More common in older adults, with rare occurrences in younger populations.
  - CML: Typically affects middle‐aged adults, though it can occur across a wide age
  - range (Table 1).

**TABLE 1:** Comparison of methods of diagnosing and classifying leukemia.

Parameter	Our center (Microscopy only)	Reference centers
Classification system	Morphology‐based (Wright‐Giemsa‐stained slides)	Flow cytometry, cytogenetics, molecular analysis
Blast percentage	Blasts ≥ 20% for acute leukemia	WHO criteria: Blasts ≥ 20%
Cell markers	Not assessed (CD markers not detectable)	CD19, CD20, CD10 (B‐ALL); CD3 (T‐ALL)
Cytogenetic testing	Not available	t(9;22), t(12;21), t(15;17) translocations
Molecular testing	Not available	Gene mutations (NPM1, FLT3, CEBPA, etc.)
Age lineage	Age‐based observations (ALL in children, etc.)	Comprehensive with flow cytometry confirmation
Smudge cells	Identified and counted for CLL diagnosis	Often used in conjunction with flow cytometry

### 2.5 Main Outcomes

The primary outcomes included age‐standardized incidence rates (ASIR) and crude incidence rates (CIR) for ALL, AML, CLL, and CML. Other variables included hematological profiles associated with specific leukemias.

### 2.6 Data Analysis

Data were reviewed for incompleteness or missing entries and then entered for analysis in statistical software packages, including IBM SPSS (Statistical Package for the Social Sciences, version 26, Chicago, IL, USA), R version 4.4.0 [28] (with packages: tidyverse, gtsummary, and apyramid), and the Joinpoint Regression Program (V.4.5.0.1). Individual observations were excluded from the analysis if more than 25% of the intended variable data were missing. Normality tests (e.g., Shapiro–Wilk test) were performed for age, and appropriate non‐parametric alternatives (e.g., Kruskal–Wallis test) were applied based on the normality of the data. For comparisons of age among different leukemia types, post hoc analysis was conducted using the Dunn test. Continuous variables were summarized as median ± interquartile range (IQR), while categorical variables (e.g., age, gender, leukemia types) were presented as frequencies or percentages. Comparisons between proportions of categorical variables were performed using the chi‐square test, unless the contingency table contained few samples, in which case Fisher's exact test was utilized.

CIR, ASIR, and estimated annual percentage change (EAPC) were calculated using the Joinpoint Regression Program (V.4.5.0.1) (Statistical Methodology and Applications Branch, Surveillance Research Program, National Cancer Institute). Briefly, the annualized CIR was estimated by dividing the total number of cases by the corresponding population at risk (estimated population size of Eritrea at the midpoint of the study period, i.e., 2015), expressed per 100 000 person‐years. The direct method was used to estimate the ASIR. This involved applying age‐specific incidence rates to a standard population (World Population Net 2014) and summing the results to obtain a weighted average, adjusted for differences in age structure. The formula for ASIR is

$$\text{ASIR}=\frac{\sum_{k=1}^{n}a_{k}\beta_{k}}{\sum_{k=1}^{n}\beta_{k}}$$

where *α*_*k*_ represents the age‐specific rate in the study population, and *β*_*k*_ represents the proportion of the standard population in the corresponding age group.

For the annualized computation of the estimated annual percentage change (EAPC), a generalized linear model (GLM) with a Gaussian distribution was employed. A regression line was fitted to the natural logarithm of the annual age‐standardized rates (ASR), expressed as y=α+βx+ε, where *x* is the year and *y* is ln(rate). From this, the EAPC was calculated as:

$$\text{EAPC}=100\times \left({\mathrm{e}}^{\beta }-1\right).$$

where *β* is the slope of the regression line, representing the annual change in the log‐transformed rates. The 95% confidence intervals (CI) for the EAPC were derived from the regression model to assess statistical significance [18].

Before interpreting the model, key assumptions were evaluated to ensure the credibility and reliability of the analysis

1. *Normality of residuals*: The residuals (differences between observed and predicted ASR values) were tested for normality using the Shapiro–Wilk test, which yielded a test statistic of 0.94 and a *p* value of 0.55. This result confirmed that the residuals followed a normal distribution. Additionally, a Q–Q plot of residuals showed that the points closely followed the diagonal line, further supporting the normality assumption.
2. *Homoscedasticity*: Homoscedasticity (constant variance of residuals) was evaluated using the Breusch‐Pagan test. The test resulted in a statistic of 0.66 and a *p* value of 0.42, indicating no evidence of heteroscedasticity. Furthermore, a residual versus fitted values plot displayed no discernible pattern, confirming that the residuals were evenly spread across all fitted values.
3. *Linearity*: The linearity assumption was assessed by visually inspecting the scatter plot of ASR against year. The data exhibited a reasonably linear relationship, supporting the use of a linear model. The model did not require logarithmic transformations, as the relationship between the variables appeared to be adequately captured without them.

The results of these diagnostic tests demonstrated that the assumptions of normality, homoscedasticity, and linearity were satisfied, confirming the appropriateness of the generalized linear model for this analysis.

## 3 Results

### 3.1 Demographic Characteristics of Patients

In this study, 372 confirmed cases of leukemia were identified in existing records. The median age (interquartile range [IQR]) at diagnosis was 48 years (IQR: 24.5–60 years), and the minimum and maximum (Min–Max) age was 2–91 years, with the male‐to‐female ratio of 210/161 or 56.6% versus 43.4%. The age‐related distribution of all leukemia was 69 (18.6%) for young patients (1–20 years), 84 (22.6%) for young adults (21–40 years), 122 (32.8%) for adults (41–60 years), and 160 (43%) for older adults (61–90 years) (Table 2). In addition, most of the patients came from the outpatient department (OPD), 167 (49.1%). Other patients were from the medical ward, 84 (24.7%) and the emergency ward accounted for 51 cases (15.0%).

**TABLE 2:** Sociodemographic characteristics of leukemic patients diagnosed in Asmara, Eritrea: 2010–2020.

Characteristic	*N* = 372^a^
Age in years	45 (23, 60)
Unknown	43
Calendar year	2015.0 (2012.0, 2017.3)
Gender	
Male	209 (57%)
Female	160 (43%)
Unknown	3
Specimen collection department	
Emergency ward	51 (16%)
Medical ward (3A)	57 (17%)
Medical ward (3B)	27 (8.3%)
OPD	167 (51%)
Pediatric	24 (7.4%)
Unknown	46
Leukemia classification	
ALL	76 (20%)
AML	41 (11%)
CLL	108 (29%)
CML	132 (35%)
Unspecified acute leukemia	15 (4.0%)
White cells count	51 (21, 133)
Unknown	310
Red cells count	2.99 (2.21, 3.62)
Unknown	312
Mean corposcular volume	91 (86, 97)
Unknown	320
Platelets count	97 (31, 269)
Unknown	310

Abbreviations: ALL, acute lymphocytic leukemia; AML, acute myelogenous leukemia; CLL, chronic lymphocytic leukemia; CML, chronic myeloid leukemia; OPD, outpatient department.

^a^ Median (IQR); *n* (%).

### 3.2 Demographic and Clinical Characteristics of Leukemia Patients by Gender in Eritrea

Table 3 presents the demographic and clinical characteristics of leukemia patients stratified by gender (male and female). The table includes data on age distribution, calendar year of diagnosis, and leukemia classification. The median age of male and female patients was similar (48 and 43 years, respectively), with no significant difference noted (*p* = 0.9). The classification of leukemia reveals proportional gender differences, with males showing a higher prevalence of Chronic Lymphocytic Leukemia (CLL) (34%) compared to females (23%), while females exhibited a higher percentage of Chronic Myeloid Leukemia (CML) (39%) compared to males (32%). Statistical analysis did not reveal significant gender differences in the overall classification of leukemia types (*p* = 0.2). This table provides an overview of the distribution of leukemia characteristics and highlights gender‐specific trends in the Eritrean population.

**TABLE 3:** Demographic and clinical characteristics of leukemia patients by gender in Eritrea.

Characteristic	*N*	Male, *N* = 209^a^	Female, *N* = 160^a^	*p* ^b^
Age (years)	329	48 (23–60)	43 (22–61)	0.9
Unknown		24	16	
Calendar year	369	2015 (2012—2018)	2016 (2012—2017)	0.6
Leukemia classification	369			0.2
ALL		43 (21%)	33 (21%)	
AML		15 (7.2%)	19 (12%)	
CLL		71 (34%)	37 (23%)	
CML		67 (32%)	63 (39%)	
AL		9 (4.3%)	6 (3.8%)	
PML		4 (1.9%)	2 (1.3%)	

^a^ Median (IQR); *n* (%).

^b^ Wilcoxon rank sum test; Fisher's exact test.

### 3.3 Comparison of Demographic Characteristics Across Leukemia Subtypes in Eritrea

Table 4 provides a detailed comparison of demographic characteristics across four leukemia subtypes: ALL, AML, CLL, and CML. Age at diagnosis showed significant variation between subtypes (*p* < 0.001), with ALL patients being the youngest (median age: 25 years) and CLL patients being the oldest (median age: 59 years). Gender distribution also differed across subtypes; males were predominant in CLL (66%) and ALL (57%), while females constituted a higher proportion in AML (56%). The calendar year of diagnosis showed no significant differences among the subtypes (*p* = 0.12). This table highlights key epidemiological differences between leukemia subtypes in Eritrea, offering insights into age‐ and gender‐related patterns in disease presentation.

**TABLE 4:** Comparison of demographic characteristics across leukemia subtypes in Eritrea.

Characteristic	*N*	ALL, *N* = 76^a^	AML, *N* = 35^a^	CLL, *N* = 108^a^	CML, *N* = 132^a^	*p* ^b^
Age (years)	329	25 (10–39)	30 (20–57)	59 (40–68)	49 (38–61)	**< 0.001**
Unknown		4	7	10	17	
Gender	369					
Male		43 (57%)	15 (44%)	71 (66%)	67 (52%)	0.2
Female		33 (43%)	19 (56%)	37 (34%)	63 (48%)	
Unknown		0	1	0	2	
Calendar year	372	2016.5 (2013–2019)	2015 (2012–2019)	2015 (2012–2017)	2015 (2012–2017)	0.12

*Note:* The significant *p* value is following Wilcoxon rank sum test are shown in bold.

^a^ Median (IQR); *n* (%).

^b^ Wilcoxon rank sum test; Fisher's exact test.

### 3.4 Age Distribution of Different Types of Leukemia

**FIGURE 2:**
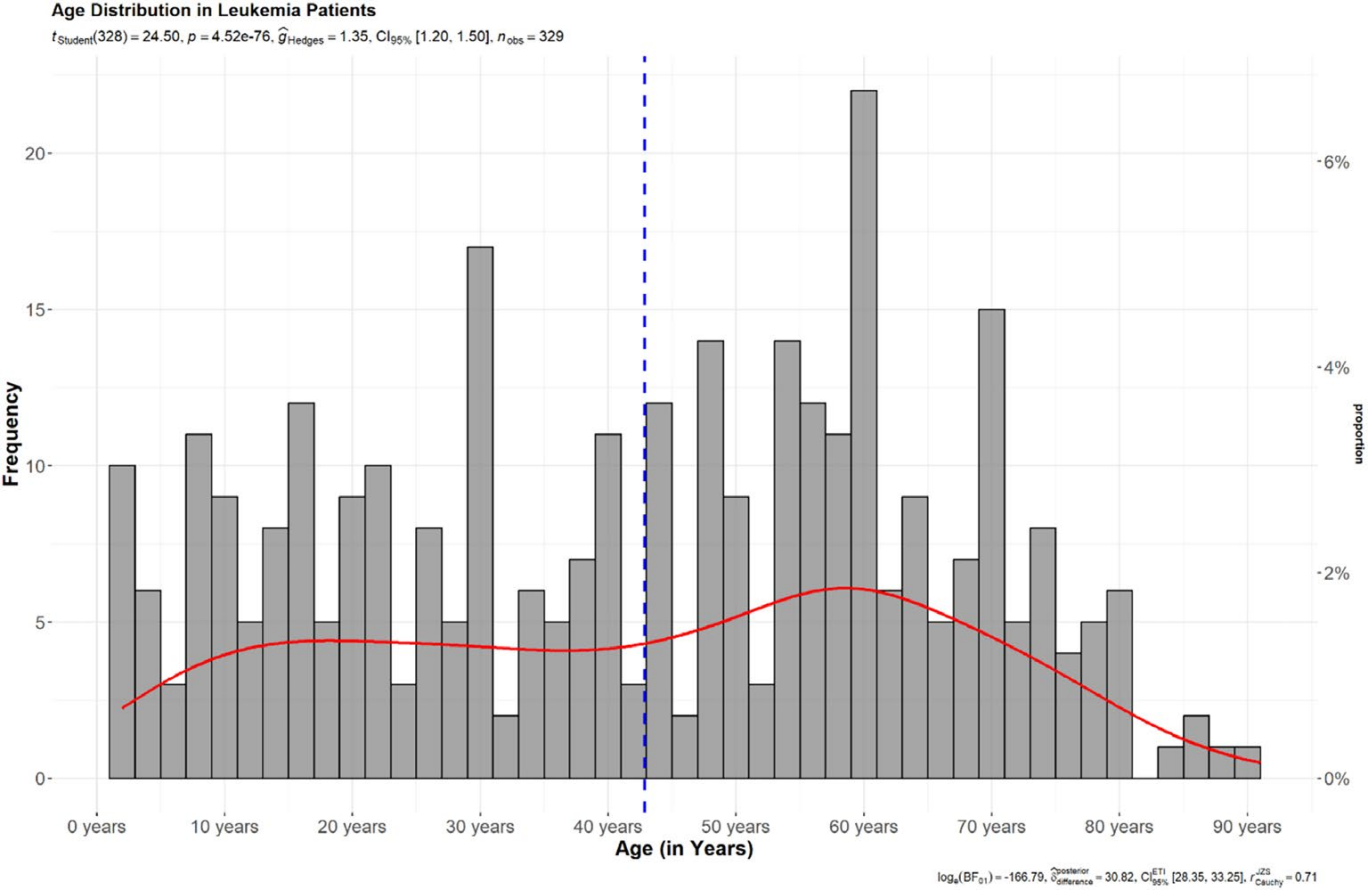
Histogram and statistical description of age among the leukemia patients in Asmara, Eritrea (2010–2020).

**FIGURE 3:**
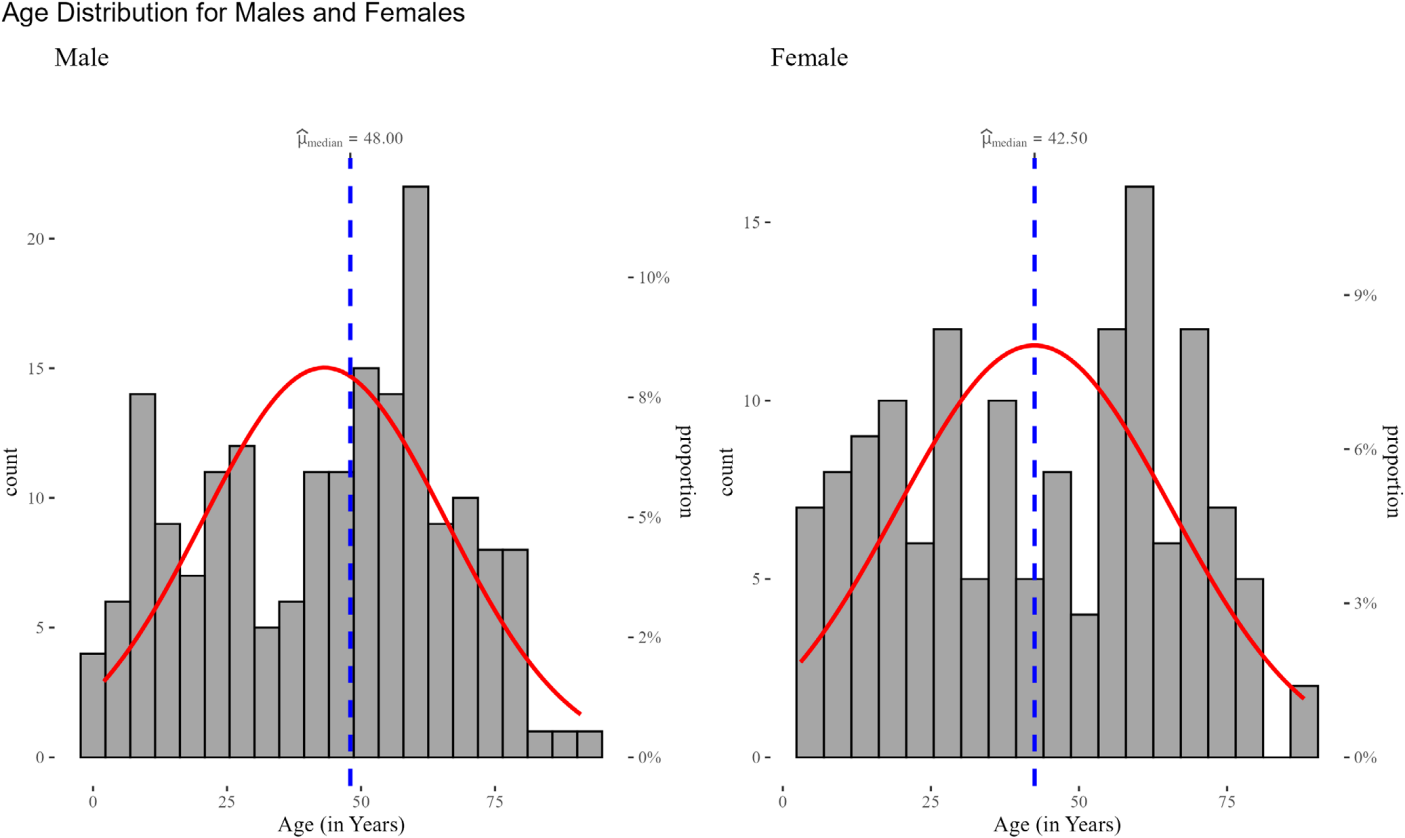
Histograms and statistical description of age stratified by gender of the leukemia patients in Asmara, Eritrea (2010–2020).

The overall age distribution of leukemia cases in this population was bimodal, showing peaks in early life and among the elderly (Figures 2 and 3). Figure 2 visualizes the age distribution of leukemia patients across the studied population. The central tendency, represented by a vertical dashed line (median age = 48 years), indicates a concentration of cases in the middle‐aged group. The fitted red curve highlights the density of cases across various ages, with peaks observed in specific decades of life, demonstrating a diverse age range. The significant statistical findings (*p* < 0.001) suggest distinct age clustering, which may inform targeted screening and treatment strategies for leukemia.

Figure 3 breaks down the age distribution of leukemia patients by gender into two histograms (male and female). The median age for males (48 years) is slightly higher than that for females (43 years), as indicated by the vertical dashed lines in each subplot. The red density curves show comparable trends between genders, with males exhibiting slightly higher frequency peaks in older age groups. Statistical analysis reveals no significant differences in the overall age distribution between genders. This comparison underscores the demographic similarities while allowing for nuanced observations related to disease burden by sex.

**FIGURE 4:**
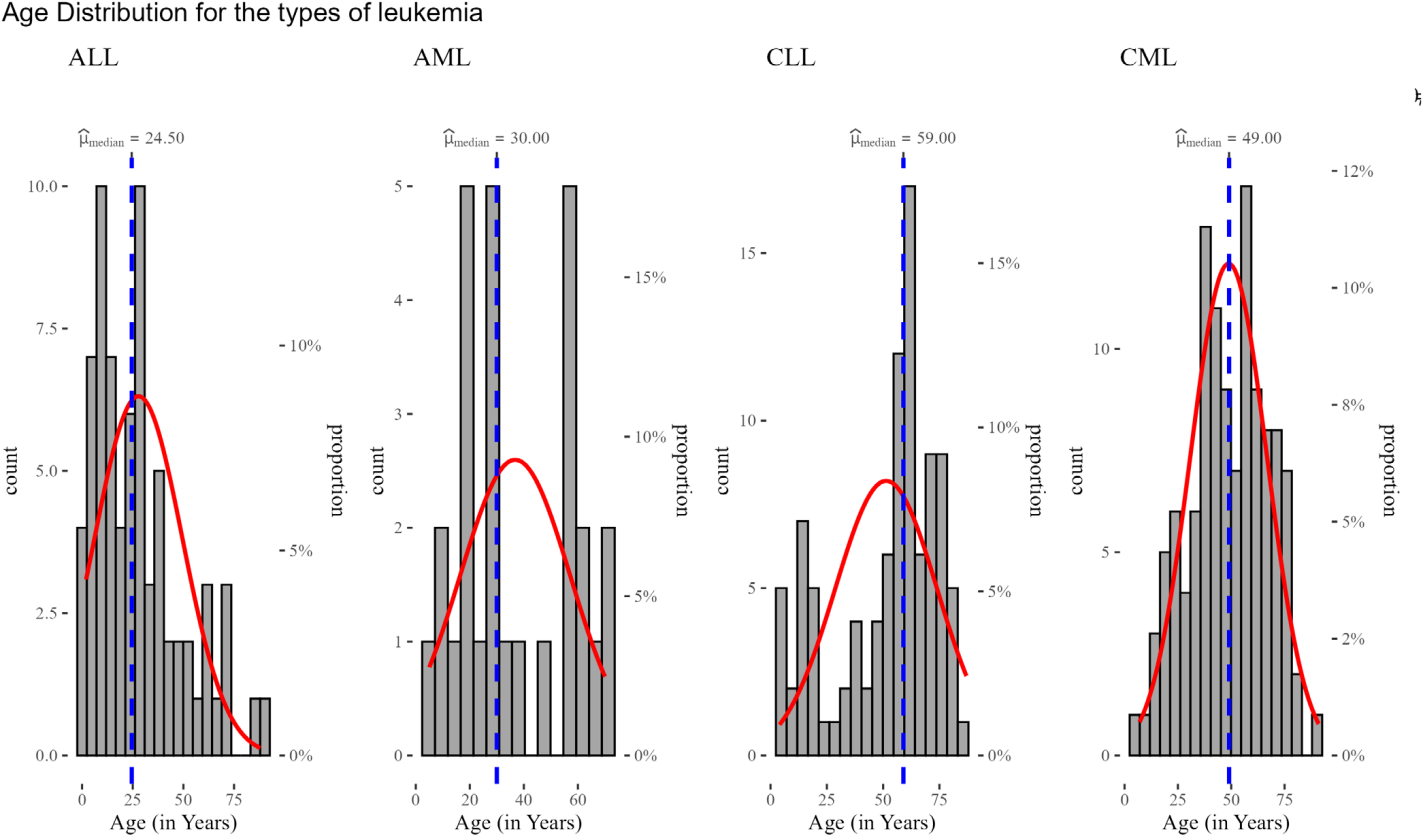
Histograms and statistical description of age stratified by leukemia types in National Health Laboratory, Eritrea (2010–2020).

This multi‐panel histogram (Figure 4) separates the age distribution by leukemia subtypes, highlighting unique age patterns for each type:

- *ALL*: Predominantly affects younger age groups (median = 25 years), with a rapid decline in frequency after early adulthood.
- *AML*: Concentrated in younger adults and middle‐aged individuals (median = 30 years).
- *CLL*: Strongly associated with older adults, with the median age peaking at 59 years.
- *CML*: Affects a broader age range, with a median of 49 years and substantial representation in middle‐aged individuals.

The red density curves and median age markers offer insights into the variability of age onset by leukemia type, with significant differences in age distribution (*p* < 0.001). This figure demonstrates the diverse epidemiological characteristics of leukemia subtypes.

**FIGURE 5:**
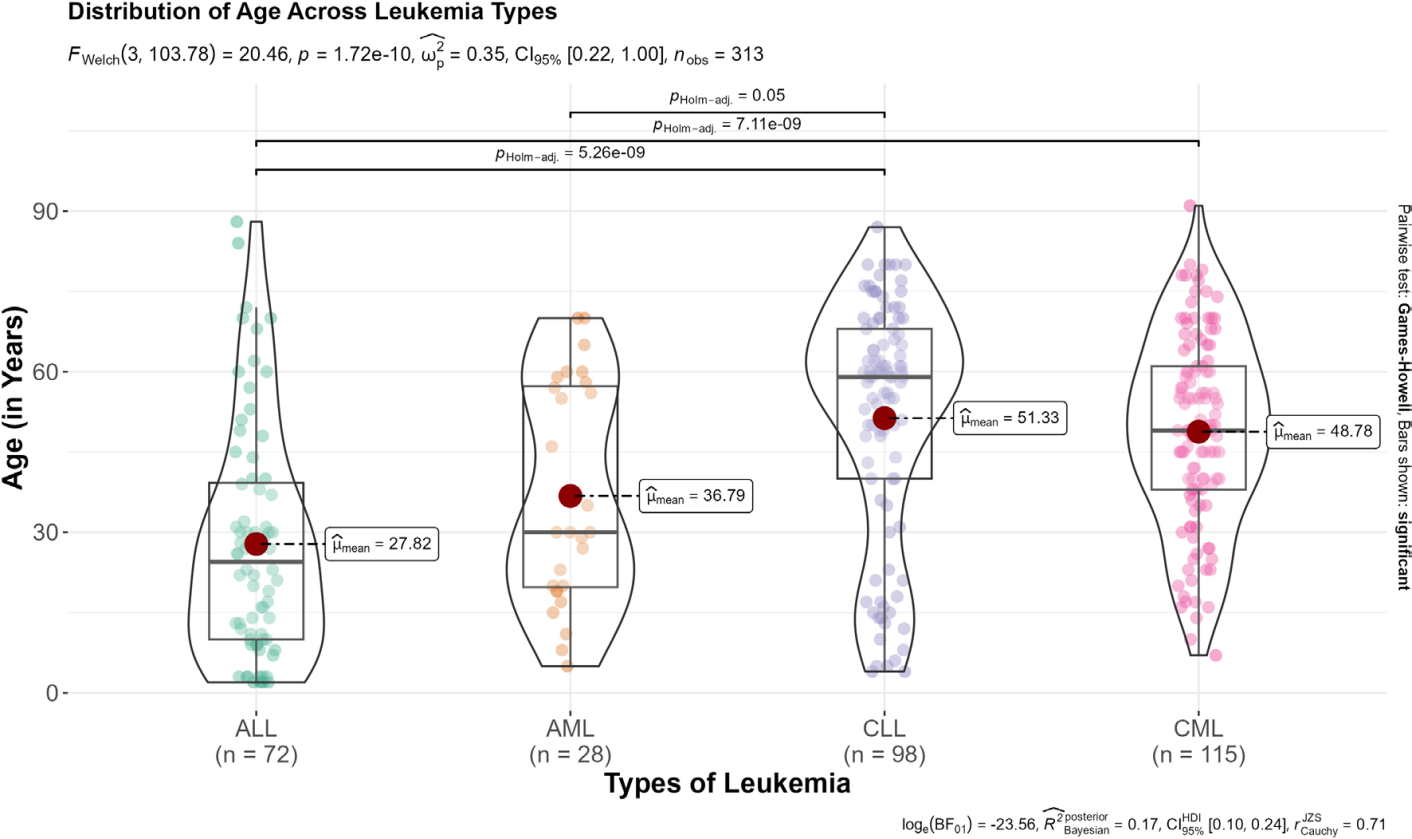
Comparison of different leukemia types by age with statistical comparison among the leukemia patients in Asmara, Eritrea (2010–2020).

Furthermore, the median (IQR) age differed significantly across different subtypes of leukemia: ALL (23.0 years, IQR: 10.0–39.0); AML (30 years, IQR: 20–56 years); CLL (59.0 years, IQR: 40.75–66.75 years); and CML (49 years, IQR: 39.25–60 years), *p* value (Kruskal–Wallis < 0.05). No sex‐specific differences were observed in median (IQR) for different types of leukemia. The post hoc test reveals that significant differences exist primarily between CLL and the other leukemia types (ALL and AML), as well as between ALL and CML. There is no evidence to suggest significant differences between ALL versus AML, AML versus CML, or CLL versus CML after Bonferroni adjustment. The results suggest that patients with CLL and CML tend to have distinct age distributions compared to other types, particularly ALL and AML. See Figure 5 for additional information.

**FIGURE 6:**
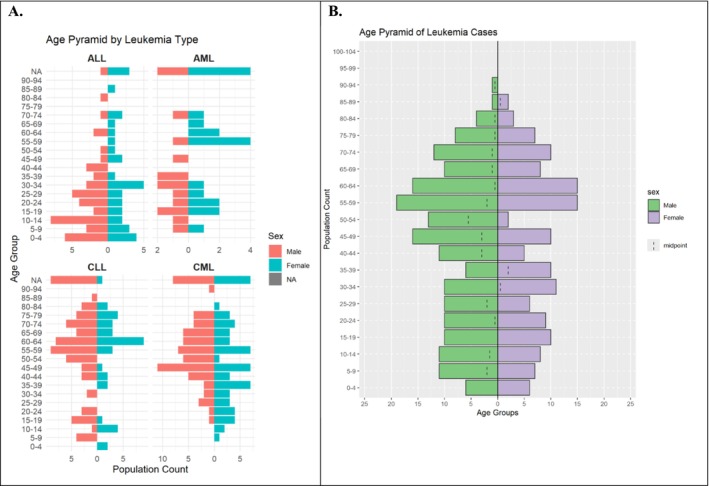
Distribution of different leukemia types across age bands (A), and overall count (B) in National Health Laboratory, 2010–2020.

The age‐sex pyramid in Figure 6 provides a detailed demographic breakdown of leukemia types (ALL, AML, CLL, and CML) by age group and gender:

- *ALL and AML*: Show a balanced male‐to‐female ratio across most age groups, with younger age groups being predominantly affected.
- *CLL*: Displays a higher prevalence in older males, particularly in the 60–64 and 65–69 age groups.
- *CML*: Shows a broader distribution, with a relatively equal representation of males and females across middle‐aged and older age groups.

### 3.5 Hematological Profiles at Diagnosis

**FIGURE 7:**
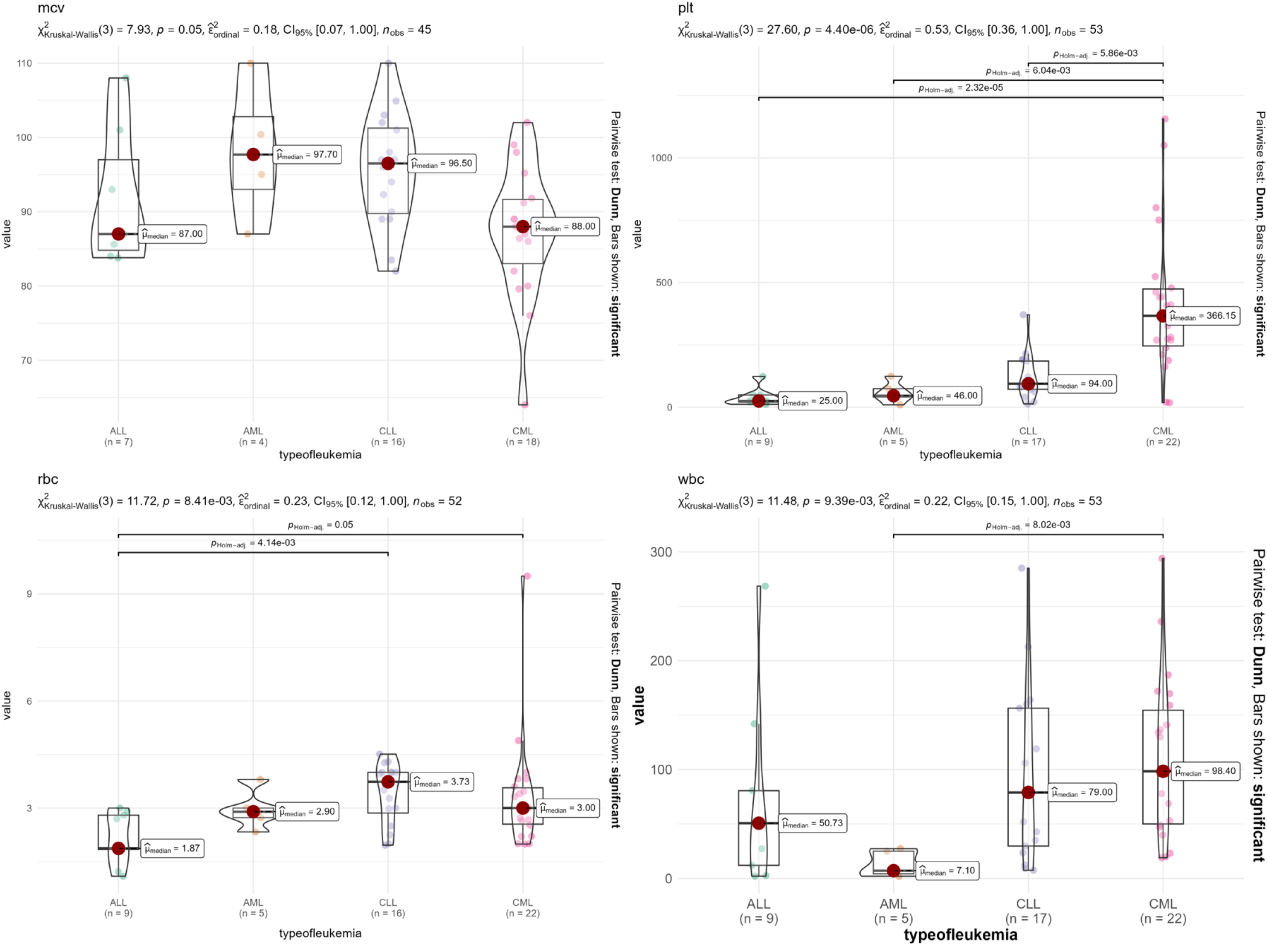
Boxplots of hematological parameters compared across different leukemia subtypes using Kruskal–Wallis *H* test and post hoc analysis using Dunn test.

A preliminary and restricted analysis to evaluate presentation at diagnosis was conducted using complete blood count (CBC) data for a selected group of patients (102 out of 369). Due to missing data, this analysis was restricted to the following timeframe: 2016, 2017, 2019, and 2020. The median (IQR) values of total white blood cell count (WBC), mean corpuscular volume (MCV), platelet count (PLTs), and hemoglobin (Hb) at diagnosis were 50.26 × 10^9^/L (IQR: 20 × 10^9^/L—134.7 × 10^9^/L); 90.6 fL (86.1–97.8 fL); 97.0 × 10^9^/L (27.5 × 10^9^/L—270.25 × 10^9^/L); and 10.0 g/dL (7.8–11.0 g/dL), respectively. Overall, 62% of the patients were anemic at diagnosis (Hb < 10 g/dL), while 51.6% were thrombocytopenic (platelet count < 100 × 10^9^/L). As seen in Figure 3, significant differences (*p* value < 0.05) in median (IQR) values were observed in disparate subtypes of leukemia. See Figure 7 for further illustration.

CML stands out with consistently significant differences in PLT, WBC, and MCV compared to other types, which may reflect unique disease mechanisms or clinical characteristics.

These findings could inform targeted diagnostic or therapeutic strategies based on hematological profiles. Further clinical and biological research is needed to elucidate the underlying causes of these differences. However, this analysis is based on a limited portion of the study sample and is therefore not generalizable.

### 3.6 Incidence of Leukemia by Gender Diagnosed in Asmara, Eritrea: 2010–2020

The overall cumulative incidence rate (CIR) was 9.22 per 100 000 persons, with an ASIR of 30.1 per 100 000 persons. Over 10 years, the estimated annual percentage change (EAPC) was −1% (95% CI: −6.1 to 6.7, *p* = 0.9), indicating no significant overall trend in leukemia incidence (Table 5).

- *Males*: CIR was 10.46 per 100 000 and ASIR was 18.3 per 100 000, with no significant temporal change (AAPC = −2.1%; *p* = 0.56).
- *Females*: CIR was 8.92 per 100 000 and ASIR was 12.06 per 100 000, with a significant decline over time (EAPC = −3.2%; *p* = 0.035).

**TABLE 5:** Incidence of leukemia by gender diagnosed in Asmara, Eritrea: 2010–2020.

Year	Male			Female			Male:Female	Both Total		
	Count	CIR	ASIR	Count	CIR	ASIR		Count	CIR	ASIR
2010	21	1.16	1.54	4	0.22	0.28	5.3:1	25	0.69	1.82
2011	18	0.10	1.33	15	0.83	1.2	1.2:1	33	0.91	2.53
2012	17	0.94	1.77	25	1.38	1.86	1:1.5	42	1.16	3.63
2013	24	1.33	2.12	12	0.67	1.01	2:1	36	0.10	3.13
2014	17	0.67	1.59	11	0.61	0.92	1.6:1	28	0.64	2.51
2015	15	0.83	1.28	13	0.72	0.88	1.2:1	28	0.78	2.16
2016	20	1.11	1.75	19	1.05	1.37	1.1:1	39	1.08	3.12
2017	24	1.33	2.09	22	1.22	1.48	1:1.1	46	1.26	3.57
2018	10	0.55	0.82	11	0.61	0.92	1:1.1	21	0.58	1.74
2019	12	0.67	1.01	17	0.94	1.22	1:1.4	29	0.80	2.23
2020	32	1.77	2.73	12	0.67	0.92	2.7:1	44	1.22	3.65
EAPC	2.1			−3.2				−0.1		
95 (CI)	−5.7 − 10.6			‐10‐4.1				−6.1‐6.7		
*p* value	0.56			0.35				0.9		
Total	210	10.46	18.03	161	8.92	12.06	1.3:1	371	9.22	30.1

Abbreviations: ASIR, age standardized incidence rate; CIR, crude incidence rate.

While males had a higher leukemia incidence, females exhibited a significant decline over the study period.

### 3.7 Crude Incidence Rate and Age‐Standardized Incidence Rates for Subtypes of Leukemia Diagnosed in Eritrea: 2010–2020

The CIR, ASIR, and EAPC for leukemia subtypes highlight variations in disease burden and trends over the study period (2010–2021) (Table 6).

- *ALL*: CIR = 2.01, ASIR = 3.87 per 100000. EAPC = 21.9% (95% CI: 3.1–44.1, *p* = 0.025), showing a significant increase in incidence.
- *AML*: CIR = 0.94, ASIR = 2.38 per 100,000. EAPC = 14.9% (*p* = 0.57), with a non‐significant upward trend.
- *CLL*: CIR = 2.94, ASIR = 15.37 per 100 000. EAPC = −2.1% (*p* = 0.65), suggesting stable or slightly declining incidence.
- *CML*: CIR = 3.61, ASIR = 24.03 per 100000. EAPC = −0.2% (*p* = 0.95), indicating no significant temporal change.

**TABLE 6:** Crude incidence rate and age‐standardized incidence rates for leukemia subtypes diagnosed in Asmara, Eritrea: 2010–2020.

Year of diagnosis	ALL		AML		CLL		CML		Overall	
	CIR	ASIR	CIR	ASIR	CIR	ASIR	CIR	ASIR	CIR	ASIR
2010	7 (0.19)	0.06	4 (0.11)	0.22	6 (0.16)	0.83	8 (0.22)	2.33	25 (0.69)	3.44
2011	2 (0.05)	0.24	0 (0)	0.00	13 (0.36)	1.31	15 (0.41)	1.23	30 (0.83)	2.78
2012	6 (0.16)	0.30	7 (0.19)	0.34	10 (0.27)	1.49	17 (0.47)	2.26	40 (1.11)	4.39
2013	8 (0.22)	0.50	4 (0.11)	0.06	14 (0.38)	2.35	11 (0.30)	2.17	37 (1.02)	5.08
2014	6 (0.16)	0.26	2 (0.05)	0.19	8 (0.22)	1.11	12 (0.33)	3.24	28 (0.77)	4.80
2015	4 (0.11)	0.05	2 (0.05)	0.19	13 (0.36)	1.60	9 (0.24)	2.15	28 (0.77)	3.99
2016	5 (0.13)	0.39	2 (0.05)	0.03	12 (0.33)	1.73	15 (0.41)	2.53	34 (0.94)	4.68
2017	9 (0.24)	0.66	3 (0.08)	0.04	14 (0.38)	2.31	14 (0.38)	2.03	40 (1.11)	5.04
2018	9 (0.24)	0.68	0 (0)	0.00	6 (0.16)	0.96	6 (0.16)	0.84	21 (0.58)	2.48
2019	5 (0.13)	0.25	7 (0.19)	0.74	7 (0.19	0.94	8 (0.22)	0.48	27 (0.75)	2.41
2020	14 (0.38)	0.48	4 (0.11)	0.57	5 (0.13)	0.74	17 (0.47)	4.77	40 (1.11)	6.56
EAPC	21.9		14.9		−2.1		−0.2		2.1	
95 (CI)	3.1–44.1		−2.3 to 38.2		−5.9 to 21		−6 to 19		−1.5 to 5.9	
*p* value	0.025		0.57		0.65		0.95		0.25	
Total	75 (2.01)	3.87	35 (0.9)	2.38	108 (2.94)	15.37	116 (3.61)	24.03	350 (9.68)	

Abbreviations: ALL, acute lymphocytic leukemia; ASIR, age standardized incidence rate; CIR, crude incidence rate; EAPC, average annual percentage change.

Overall, leukemia incidence was highest for CML, while ALL showed a statistically significant increase.

### 3.8 Crude Incidence Rate and Age‐Standardized Incidence Rates for Leukemia Across 10‐Year Age Bands: 2010–2021

The CIR and ASIR varied across age bands. The highest CIR was observed in individuals aged 70–79 years (5.05 per 100 000), while the highest ASIR was in the 50–59 years age group (7.16 per 100 000). Leukemia incidence was lowest in children under 10 years (CIR: 0.51; ASIR: 1.02 per 100 000). A notable increase in incidence was seen in middle‐aged and older adults, with a decline in ASIR in those over 80 years. Full details are presented in Table 7.

**TABLE 7:** Crude incidence rate and age‐standardized incidence rates for leukemia across 10‐year age bands: 2010–2020.

Year	< 10	10–19	20–29	30–39	40–49	50–59	60–69	70–79	> 80
	CIR/ASIR	CIR/ASIR	CIR/ASIR	CIR/ASIR	CIR/ASIR	CIR/ASIR	CIR/ASIR	CIR/ASIR	CIR/AISR
2010	0.51/0.15	1.01/0.31	0/0	0.42/0.12	0/0	3.89/0.68	0.79/0.12	5.05/0.3	3.96/0.14
2011	0.61/0.18	0.23/0.08	1.04/0.22	1.05/0.18	4.56/0.7	3.33/0.44	6.34/0.52	2.52/0.21	0/0
2012	0.4/0.07	0.56/0.12	0.52/0.12	3.15/0.49	6.32/0.79	7.77/1.24	3.97/0.32	3.79/0.19	7.93/0.29
2013	0/0	0.11/0.04	1.57/0.39	1.05/0.18	3.51/0.72	3.89/0.68	9.52/0.85	6.32/0.27	0/0
2014	0.31/0.08	0.33/0.08	0/0	1.05/0.25	1.40/0.27	4.44/0.8	10.31/0.84	3.79/0.19	0/0
2015	0.31/0.08	0.68/0.12	1.04/0.22	1.47/0.31	1.75/0.36	1.66/0.57	3.96/0.2	1.26/0.3	0/0
2016	0.41/0.11	0.90/0.2	1.22/0.23	1.47/0.25	1.40/0.36	3.331/0.44	8.72/0.87	13.9/0.66	0/0
2017	0.71/0.18	1.12/0.24	0.87/0.17	2.73/0.43	2.45/0.54	5.00/0.91	2.38/0.22	12.63/0.67	15.87/0.2
2018	0/0	0.34/0.08	1.22/0.28	0.21/0.06	1.40/0.27	2.78/0.32	0.79/0.12	7.58/0.26	19.8/0.35
2019	0.41/0.07	0.56/0.12	1.74/0.39	1.05/0.18	1.40/0.17	4.99/0.53	3.17/0.33	3.79/0.3	3.97/0.21
2020	0.51/0.18	0.45/0.15	0.7/0.23	0.63/0.18	3.51/0.9	2.78/0.55	6.35/0.9	3.79/0.31	7.93/0.25

Abbreviations: ASIR, age adjusted incidence rates; CIR, crude incidence rates.

## 4 Discussion

The results of this retrospective analysis demonstrate a substantial burden of leukemia in Eritrea. The main finding of our study is that the cumulative (2010–2021) CIR and ASIR of leukemia in this population were 9.22 per 100 000 and 30.1 per 100 000, respectively, with a male‐to‐female (M/F) ratio of 1.3:1. The incidence of leukemia was higher than previous estimates [16, 23, 29]. However, the lack of nationally representative data in most similar settings limits the generalizability of these findings [22, 29]. Although the ASIR estimates are comparable to those in a recent report (3.99 per 100 000 in 2015), they are lower than estimates from Ethiopia (6.5 per 100 000) [30]. The higher incidence of leukemia in males is consistent with current global reports [13] and has been associated with greater exposure to risk factors such as smoking and occupational hazards [31].

Disaggregation of data by age showed a higher incidence of acute leukemias (ALL and AML) in younger patients and a higher incidence of chronic leukemias (CML and CLL) in older patients. The < 10 age group exhibited low CIR/ASIR consistently across years (e.g., 0.51/0.15 in 2010, 0.71/0.18 in 2017), whereas globally, this age group often sees a peak in leukemia incidence, particularly acute lymphoblastic leukemia (ALL) [32]. Conversely, the 70–79 and > 80 age groups exhibited surprisingly high rates (e.g., > 80 in 2017: CIR 15.87, ASIR 0.2). While an increase with age is expected for chronic leukemias (e.g., CLL) [33], the rates for older adults here are disproportionately high relative to younger age groups. Dramatic year‐to‐year changes in CIR/ASIR, especially in older age groups, were observed: for example, the 70–79 age group showed a CIR spike in 2016 (13.9) and 2017 (12.63), followed by a significant drop in 2018 (7.58). Similarly, in individuals older than 80 years, the CIR increased from 7.93 in 2016 to 19.8 in 2018 but then dropped to 3.97 in 2019. Such variability suggests possible inconsistencies in diagnosis, reporting, or case ascertainment. The CIR/ASIR for the 20–29, 30–39, and 40–49 age groups was unexpectedly low in many years (e.g., 0/0 for 20–29 in 2014; 0.21/0.06 for 30–39 in 2018). This is peculiar because these age groups often see cases of AML and CML globally [34].

The median age of patients with CML (49 years, IQR: 39.25–60 years) was strikingly similar to that reported in a clinical trial of imatinib (a tyrosine kinase inhibitor) from high‐income countries (HICs) [35]. This age distribution may reflect the age structure of the population in Eritrea or other unidentified biases. In contrast, ALL has been described as the least common type overall, with peak incidence occurring at 3–5 years of age. Although CML is rarely diagnosed in the region [36], we reported a relatively high prevalence (132 cases, 35%). High levels of CML have previously been reported in Morocco and Kenya [37, 38].

Further assessment of the frequency of disparate subtypes of leukemia in this population uncovered some remarkable findings. In terms of proportions, we found that the overall all‐age distributions were as follows: ALL (20.1%), AML (11.0%), CLL (29.0%), and CML (35.4%). In contrast, a different distribution was observed globally and in the region. In Northwest Ethiopia, the following distribution was observed: CLL (61.1%), ALL (23.2%), CML (6.3%), AML (4.2%), and other hematological malignancies (5.3%) [36, 39]. According to Dong et al., CLL and AML were the predominant subtypes in high‐SDI countries located in Europe and North America [40]. In contrast, CML was rarely diagnosed. Relative to these reports, our results are highly unusual, considering the disproportionate diagnosis of CML. Nevertheless, we must emphasize that the proportions of disparate leukemia subtypes are highly heterogeneous in between‐country comparisons, and the underlying reasons for these disparities remain poorly understood.

A further difference observed in this population was the age at diagnosis of ALL (23.0 years vs. 5.0 years in Egypt) [41]; CLL (59.0 years vs. 59 years in Nigeria and 70 years in the USA) [42]; and CML (49 years vs. 34 years in Rwanda). Of note, the low median age for CML and its predominance in this population is unusual. In general, the dominant risk factors for CML are aging and exposure to radiation [43]. Indeed, past reports suggest that the incidence of CML correlates strongly with advancing age, peaking at 10.3 cases per 100 000 among individuals aged 80–84 years, with a frequent age at diagnosis of 65–74 years [43]. More recently, Hoglund et al. argued that geographic disparities in ASIRs of CML could partly be attributed to methodological factors such as the overrepresentation of referral patients in some registries [44]. Others have argued that the advent of tyrosine kinase inhibitors (TKIs) has transformed CML from a fatal disease to a disorder compatible with a normal lifespan, thereby increasing its prevalence [45]. We do not believe this is the case in low‐ and middle‐income countries (LMICs). However, this argument, together with existing demographic trends in HICs, may explain the high burden of CML in these regions. Altogether, we must concede that the unusual distribution of leukemia subtypes or age at diagnosis is difficult to explain and warrants further investigation.

Importantly, we noted that the incidence of AML, CLL, and CML remained stable over the years. This contrasts with recent reports that have uncovered increases in CLL (0.46% per year from 1990 to 2017) and decreases in CML in most regions [40]. In another unusual finding, we noted that the estimated annual percentage change (EAPC) associated with ALL increased significantly over the years. In contrast, a recent report noted that the ASIR associated with ALL experienced a subtle decrease from 1990 to 2017 (EAPC = −0.08, 95% CI: −0.15, −0.02), with the greatest decrease detected in Ghana (EAPC = −5.18, 95% CI: −6.23, −4) [42]. Altogether, the increasing ASIR in ALL may be caused by environmental factors and requires further research.

Eritrea's unique geographic, environmental, and socio‐economic conditions could provide important context for understanding the observed leukemia trends. The increasing ASIR in ALL may be caused by environmental or occupational exposures that warrant further investigation. Eritrea's agrarian society raises the possibility of exposure to pesticides, herbicides, and fertilizers, which have been linked to leukemia pathogenesis globally. Additionally, prolonged exposure to naturally occurring toxins in water or soil, such as aflatoxins or heavy metals, could contribute to hematological malignancies. Occupational exposures, particularly in Eritrea's mining sector, could expose workers to carcinogenic agents like benzene, arsenic, and silica dust, all of which are strongly associated with leukemia. Informal or unregulated work settings may further amplify these risks. Furthermore, chronic infections, such as Epstein–Barr virus (EBV) or human T‐cell leukemia virus (HTLV), which are more prevalent in certain regions, may also play a role. Other factors, including malnutrition, immune suppression, urbanization, and pollution, might compound these risks. While global trends show decreases in ALL and CML in certain regions, the increasing ASIR for ALL in Eritrea could also be influenced by genetic predispositions or unique epigenetic modifications resulting from environmental exposures. These observations emphasize the need for detailed epidemiological and case–control studies, including environmental monitoring of pesticide residues and heavy metals, occupational studies on high‐risk industries, and genetic research to identify potential predispositions.

The absence of data has not deterred experts from speculating on the potential drivers of leukemia epidemiology in Africa. In the past, disparities in cancer burden or patterns of distribution and trends have been explained by specific risk factor dynamics, underdiagnosis, under‐registration, misdiagnosis, or changes in classification protocols [46]. Underdiagnosis is mostly a by‐product of well‐documented deficiencies in laboratory systems and health delivery components, such as the management of health information [47]. Emphasizing this point, authors of a recent Global Burden of Disease (GBD) report noted that < 10% of the population in Africa and other LMICs in Asia and South America are covered by high‐quality cancer registries [46]. According to Lam et al., some types of cancers are poorly represented in data from some regions [48]. Underreporting (the deficit between actual and reported incidences) is more pronounced in malignancies such as acute leukemias, neuroblastomas, and bone tumors due to the requirement of advanced diagnostic techniques (e.g., flow cytometry markers, immunophenotyping, immunohistopathology, and advanced imaging technologies such as positron emission tomography [PET] scans) [48]. Altogether, the problem of underreporting and the lack of population‐based cancer registries (PBCRs) means that estimates of cancer burden rely heavily on covariate selection in models and regional patterns [46]. Much of what is described here is true for Eritrea. As previously noted, the concentration of oncologic and diagnostic infrastructure in Asmara, Zoba‐Maekel, limits access to cancer care in the country. This problem is potentially compounded by knowledge deficits among health workers and poor referrals to specialized/reference hospitals.

A detailed analysis of the etiologies behind the observed variation in leukemia incidence across countries in sub‐Saharan Africa (SSA) goes beyond the scope of this analysis. However, multiple factors from studies in HICs and non‐African populations have been invoked. For example, the diverse incidence of leukemias across GBD super‐regions is driven by between‐country disparities in occupational exposures to putative risk factors such as aging and environmental hazards (e.g., benzene, formaldehyde, and other persistent organic pollutants [POPs]) [34, 49, 50]; therapeutic exposures (e.g., alkylating agents and topoisomerase II inhibitors); prenatal and postnatal exposure to ionizing radiation (particularly X‐rays) [32]; herbicides or pesticides; obesity (body mass index [BMI] ≥ 30 kg/m^2^); smoking or tobacco use; and birth weight [51, 52]. The role these factors currently play in the observed increase in ALL is unknown. However, the difference in prevalence and ASIRs between males and females is generally attributed to differential exposures to risk factors such as smoking (a predominantly male activity) and obesity [32]. Interestingly, a recent report noted that in 2019, ALL and AML were the leading causes of leukemia attributable to occupational risk‐related disability‐adjusted life years (DALYs) and death rates [52].

The development of leukemia has also been partly ascribed to a number of syndromes whose epidemiology in SSA is poorly understood, including genetic syndromes (e.g., Down syndrome, Li‐Fraumeni syndrome, neurofibromatosis, Fanconi anemia, and Bloom syndrome) [53]. Other factors implicated in leukemogenesis include acquired or inherited genetic and epigenetic mutations, such as CLL (chromosome 13q deletion [del(13q)], hyperdiploidy) [41]; CML (9:22 translocation [Philadelphia chromosome] [q34; q11.2] or BCR‐ABL fusion); and AML (t(15;17), t(8;21), inv. (16), or t(16;16)), among others. Research on the connection between this panoply of risk factors and leukemia in SSA remains inadequate.

Literature currently supports a link between AML and the Human Development Index (HDI)—a composite measure of life expectancy, education, and standard of living. In general, countries with high HDI tend to report higher ASIRs for AML [34]. The reasons behind this phenomenon remain unexplored but may be related to genetics [54]. Others have implicated occupational exposures such as benzene, organic solvents (including gasoline), cigarette smoking (≥ 20 pack‐years, relative risk 1.34), and obesity (best documented in women) [34]. In contrast, exposure to carcinogens appears to be a major driver of AML in countries with low HDI. However, whether exposures to carcinogens have a high attributable risk for AML in Eritrea is difficult to ascertain due to the lack of data. Even in the absence of data on exposures, countries are still advised to promote relevant public health interventions.

Unlike AML and other leukemias, ALL ASIR is not patterned by HDI. However, most of the exposures associated with ALL are similar to those of AML—radiation (for childhood ALL) and chemical exposures [40]. Regarding radiation, practices such as X‐ray pelvimetry during pregnancy are known to elevate risks [40]. The prevalence of this practice in clinics in Eritrea or the region is poorly documented. In more recent times, some researchers have espoused an infectious disease etiology for pediatric leukemias such as ALL and AML—Greaves' “delayed‐infection hypothesis” [55] and Kinlen's “population‐mixing hypothesis” [56]. Data supporting these hypotheses remain inconclusive [55]. However, researchers have argued that research on these factors should be promoted [55]. In much of Africa, for example, the infectious disease burden remains high, and a possible link between specific pathogens and ALL should be a concern.

As previously mentioned, the diagnosis of CML was higher relative to its proportion in other populations. Globally, ASIR of CML tends to vary between countries, and unlike specific leukemias, it is not patterned by HDI. The current understanding of its etiology remains limited, though radiation exposure has been linked to its development [40]. In the literature, the most important risk factor for CML is age—peaking at 10.3 cases per 100 000 among individuals aged 80–84 years [43]. In this cohort, CML ASIR was higher relative to other leukemias—an outcome that is difficult to explain—and women tended to be younger. In the past, geographic disparities in ASIRs of CML were explained with reference to mundane factors such as the overrepresentation of referral patients in some cancer registries [51]. Furthermore, some have suggested that the advent of tyrosine kinase inhibitors (TKIs) has transformed CML from a fatal disease to a disorder compatible with a normal lifespan, thereby increasing its prevalence [40]. We do not believe this is the case in Eritrea. In the absence of information on the impact of existing treatment or cancer prevention strategies within and between countries, these arguments demonstrate the difficulties associated with attempts to explain trends.

In CLL, aging and family history have been described as some of the most important risk factors. For reasons that are poorly understood, reports indicate that ASIR for CLL more than doubled between 1990 and 2017 [40]. In this study, ASIR was high, but EAPC was stable. The stable EAPCs suggest that epidemiological and sociodemographic transitions play a minimal role in CLL burden in Eritrea. Although the etiology of CLL is poorly understood, some studies have suggested that environmental factors may play a role in CLL pathogenesis. This conclusion is generally supported by data from large‐scale studies such as the InterLymph studies, which demonstrated a potential association between CLL and increasing height, hepatitis C seropositivity, residential or occupational history on a farm, and occupational history as a hairdresser [57].

Therefore, it follows that an overview of the literature supports the fact that very little is known about the etiological factors driving leukemia incidence or the molecular epidemiology of leukemias in most countries in SSA. The need for such investigations in Eritrea is therefore apparent.

## 5 Conclusion and Recommendation

Leukemia burden in Eritrea appears to be high. However, due to underreporting and underdiagnosis, it is our belief that the true burden of leukemia is likely higher. Therefore, there is a need for capacity building (training and improvement in laboratory infrastructure), decentralisation of existing services to higher‐tier facilities (referral hospitals) in other sub‐zones; and establishment of a cancer registry. In the last two decades, advances in laboratory techniques and technologies (next‐generation sequencing—cancer genomics, immunophenotyping or flow cytometry, cytogenetics, among others) have greatly improved the diagnosis and management of leukemias. Although a daunting or perhaps impossible task in LMICs, the adoption, at scale, of some of these technologies in laboratories in Eritrea should be explored. Research is also required to understand other aspects of leukemia in Eritrea—mortality, treatment outcomes, among others.

## Author Contributions

Conceptualization: D.M.A. and E.G. Data curation: D.M.A., E.G., E.G., G.E.Z., R.K., A.T., S.N., T.D., and S.M. Formal analysis: S.T.M. and O.O.A. Investigation: S.T.M., D.M.A., E.G., and O.O.A. Methodology: S.T.M., D.M.A., E.G., E.G., G.E.Z., R.K., A.T., S.N., and T.D. Project administration: D.M.A. and E.G. Writing – original draft: D.M.A., E.G., and O.O.A. Writing – review and editing: S.T.M., D.M.A., O.O.A., and E.G. All authors read and approved the final manuscript.

## Ethics Statement

The study was conducted in accordance with the Declaration of Helsinki and approved by the Health Research Ethics and Protocol Review Committee of the Eritrean Ministry of Health. Informed consent was waived for this study because it involved the use of retrospective, anonymized patient records, and obtaining individual consent was not feasible. This waiver is consistent with the World Medical Association (WMA) Declaration of Helsinki (*Paragraph 32*), which allows for the waiver of consent in cases where the research involves anonymized data and obtaining consent would be impractical.

## Conflicts of Interest

The authors declare no conflicts of interest.

## Data Availability

The data used to generate the results of this manuscript is available upon a reasonable request from the corresponding author.
